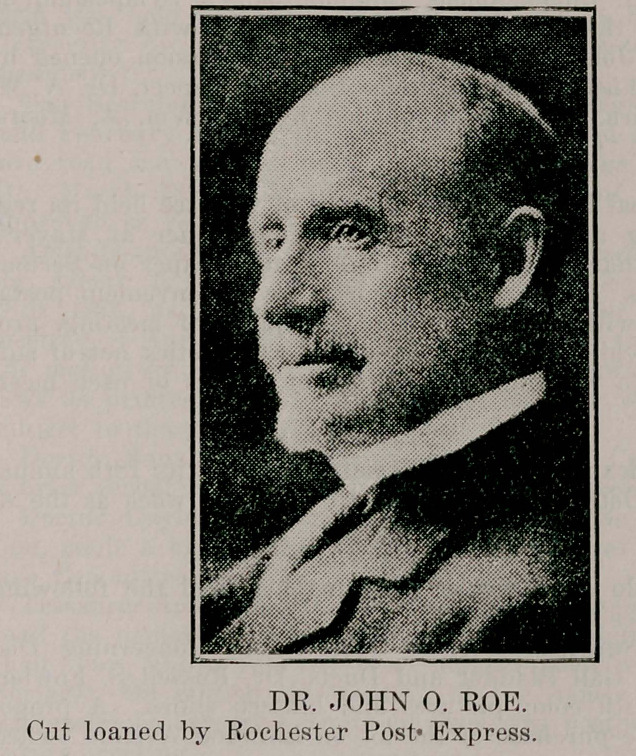# Society Meetings

**Published:** 1916-05

**Authors:** 


### SOCIETY MEETINGS

---

**Brief reports and announcements of meetings of societies of Western New York, and those of general scope, are requested from Secretaries. Copy should be on hand the fifteenth of the month. Full transactions will be published at cost of composition.**

---

**DOCTOR: Is your Society properly represented here? If not, please bring our standing announcement to the attention of the members.**

---

**The National Association for the Study and Prevention of Tuberculosis** will meet in Washington May 11-12. Papers, technical and sociologic, and clinics, will be given. A general invitation is extended. Those wishing to join the Association should apply to Dr. Charles J. Hatfield, Executive Secretary, 105 E. 22d, N. Y.

**Rochester Academy of Medicine.** Section IV. met April 12, Dr. Geo. W. Goler presenting a paper on Prophylaxis of Typhoid Fever; Dr. Joseph R. Culkin on Diagnosis and Prognosis of Typhoid.

A special memorial meeting in honor of the late Dr. John O. Roe, was held March 29. The President, Dr. Joseph R. Culkin, gave an introductory address; Dr. Eugene H. Howard read a memorial prepared by a special committee; Dr. Bradford A. Richards read a paper on the Scientific Contributions of John O. Roe, and Dr. Wendell C. Phillips of N. Y. came especially to pay a tribute to Dr. Roe. A portrait of Dr. Roe, presented by his family, was accepted by the Academy. Dr. Richard's paper was illustrated by lantern slides of some of Dr. Roe's results in rhinoplasty.

DR. JOHN O. ROE.
Cut loaned by Rochester Post Express.

---

**Eighth District Dental Society of N. Y.** The annual meeting was held at the University Club of Buffalo, April 12. Dr. Karl G. Knoche of Chicago read a paper and gave a clinic on Removable Bridge Work. Officers were elected as follows: President, M. Burton Eschelman, Buffalo; Vice-President, L. L. Mulcahy, Batavia; Secretary, Lowell Childs, Springville; Treasurer, F. A. Ballachey, Buffalo.

**The Medical Society of the State of N. Y.** meets at Saratoga, May 16-18.

---

**University of Buffalo Alumni Association.** The annual meeting of the House of Delegates was held March 22. Officers were elected as follows: President, Richard F. Morgan, Pharm. D. '97; Vice-President, D. H. Squire, D. D. S., '93; Secretary, Julian Park of the Faculty of the Arts Dept.; Treasurer, A. Glenn Bartholomew, LL.B., '03.

---

**The Binghamton and the Elmira Academy of Medicine** held a joint meeting at the rooms of the latter, April 18. The program was as follows: 1. The Present Status of Physical Therapeutics. Dr. J. C. Fisher, Elmira. Discussion opened by Dr. L. H. Quackenbush, Binghamton. 2. Symposium on Diseases of the Stomach. Medical Aspect with Roentgengraphs, Dr. John A. Bennett, Elmira. Discussion opened by Dr. H. I. Johnston, Binghamton. Surgical Aspect, Dr. A. W. Booth, Elmira. Discussion opened by Dr. Wm. A. Moore, Binghamton. Collation.

---

**The Medical Society** of **the County of Genesee** held its regular meeting at Batavia, April 5, after dinner at Mayer's cafe. Dr. Charles Bentz of Buffalo read a paper on Serums and Vaccines. (Note: This Society uses a convenient postal card blank with spaces for time and place of meeting, program, etc., which is to be commended to societies not of sufficient size to warrant the printing of notices of each meeting.)

---

**The American Proctologic Society** will hold its 18th annual meeting at Detroit, June 12 and 13, the same week as the A. M. A.

---

**The Buffalo Academy of Medicine** has held the following meetings since last noted:

April 5, Surgical Section; Observations Concerning Diseases of the Gall Bladder and Ducts, Dr. Russell S. Fowler, Brooklyn, with color pictures and lantern slides. A proposition for the purchase of the lot on Linwood Avenue owned by the Academy was received but was not accepted, although it showed that the investment had been a wise one.

April 12, Medical Section; Colloid Chemic Phenomena in the Living Cell, G. H. A. Clowes, Ph. D.; Roentgen Diagnosis of Gastro-enteric Diseases (lantern slides) Dr. Lester Levyn. (Note: We expect to publish a summary of Dr. Fowler's remarks, Dr. Levyn's paper and, later, a series embodying the substance of Dr. Clowes' talks on physical chemistry.)

April 19, Section of Obstetrics; Results of Routine Study of the Placenta, Dr. J. Morris Slemons, New Haven.

---

**A National Association of Clinical Laboratories** has been framed at a preliminary meeting in Chicago, and an organization meeting will be held in Detroit, June 12. It is to be hoped that all members of the profession interested in this field will attend the meeting. We understand that it is desired that those not necessarily connected with established laboratories and not limited to laboratory work shall attend, in order that the new organization may co-operate with clinicians and extend its usefulness along broad lines.

---

Regular meeting of the **Medical Society of the County of Erie** was held on April 17, at 8.30 p. m. in Alumni Hall, University of Buffalo, 24 High Street. President Barrows presided.

The Secretary read the minutes of the regular meeting held February 28th, 1916, which were approved as read. He also read the minutes of the Council meetings held March 7th, March 13th and April 11th, 1916, all of which were approved as read.

Amendments to the By-laws, which were presented by Doctor Albert T. Lytle at the regular meeting held February 28th, 1916, had been printed in the notice for the regular meeting of April 17th, 1916, and were read by the Secretary. On motion of Doctor Gaylord, the amendments to the By-laws as printed in the notice of the meeting were adopted subject to the approval of the State Society.

Doctor Bonnar, chairman of the Board of Censors, made a brief verbal report on the work of the Censors.

Doctor Gaylord, chairman of the Committee on Legislation, made a brief verbal report on the work of the Legislative Committee.

Treasurer Lytle stated that the By-laws required him to read the names of those members who were delinquent with their dues, unless relieved from making such report by the Society. On motion the reading of the names of members who were in arrears with their dues was postponed until a future meeting.

The Secretary stated that an invitation had been extended by the Buffalo Historical Society to the Medical Society of the County of Erie to attend a meeting in memory of Doctor Ernest Wende and Doctor Roswell Park to be held at the Historical Society Building on Tuesday evening, April 18th, 1916. At this meeting Mr. Adelbert Moot will speak on the life and work of Doctor Wende, and Doctor Charles G. Stockton will present a paper on Doctor Park. On motion of Doctor Lytle, seconded by Doctor Mann, President Barrows and Secretary Gram were designated to officially represent the Society at this Memorial Meeting.

Doctor Jacobs, chairman of the Committee on Membership, presented the names of the following applicants and moved that they be elected to membership. The motion was seconded and each applicant was separately voted upon and elected as follows:

Reinstatement of Doctor Irving R. Johnson, 403 Delaware Avenue, Buffalo.

Election to membership of Doctor William K. O'Callahan, 620 Niagara Street, Buffalo.

Election to membership of Doctor Wilfred H. Baines, 1273 Abbott Road, Buffalo.

Election to membership of Doctor Henry D. Abbott, 1355 Abbott Road, Buffalo, by transfer from the Hudson County Medical Society.

Reinstatement of Doctor Lawrence Hendee, 346 Elmwood Avenue, Buffalo.

This concluded the business part of the program.

Doctor Harvey R. Gaylord, director of the New York State Institute for the Study of Malignant Diseases, then delivered a popular lecture on the work of the Cancer Laboratory, located in the State Institute at 113 High Street, Buffalo. This lecture was accompanied by lantern slides and showed in a general manner the work as it is conducted and what has thus far been accomplished. At the close of the lecture a motion by Doctor McKee seconded by Doctor Mann, a hearty vote of thanks was given to Doctor Gaylord.

The Society then became the guests of the State Institute, where it adjourned in a body, to make a complete inspection of the various features of the Institute, such as laboratory, hospital and research work. The various activities were explained to the visiting physicians by Doctor Burton T. Simpson, Department of Pathology; Drs. G. H. A. Clowes and F. West, Department of Chemistry; M. C. Marsh, Department of Biology; T. F. Cooke, Department of X-Ray and Radium; Dr. J. A. P. Millett and Dr. Herbert Bauckus, Clinical Work in the hospital, and Dr. B. F. Schreiner, the activities of the Dispensary Service.

At the conclusion of this inspection a splendid collation was served.

How much the members appreciated this privilege was shown by one of the largest attendance of members at any meeting for a considerable period.—Franklin Gram, Sec'y.

## Figures and Tables

**Figure f1:**